# Relationship between cognitive dysfunction and the promoter methylation of PER1 and CRY1 in patients with cerebral small vessel disease

**DOI:** 10.3389/fnagi.2023.1174541

**Published:** 2023-05-24

**Authors:** Yiwen Xu, Yugang Wang, Yi Jiang, Mengqian Liu, Wen Zhong, Zhonglin Ge, Zhichao Sun, Xiaozhu Shen

**Affiliations:** ^1^Department of Geriatrics, Lianyungang Hospital Affiliated to Jiangsu University (Lianyungang Second People’s Hospital), Lianyungang, China; ^2^Department of Neurology, The First People’s Hospital of XianYang, XianYang, China; ^3^Department of Geriatrics, Lianyungang Hospital Affiliated to Bengbu University (Lianyungang Second People’s Hospital), Lianyungang, China; ^4^Department of Neurology, Lianyungang Second People′s Hospital, Lianyungang, China; ^5^Department of Pathology, Lianyungang Second People′s Hospital, Lianyungang, China

**Keywords:** clock genes, PER1, CRY1, methylation, cognitive dysfunction, cerebral small vessel disease, aging

## Abstract

**Background and purpose:**

The prevalence of cerebral small vessel disease (CSVD) is increasing due to the accelerating global aging process, resulting in a substantial burden on all countries, as cognitive dysfunction associated with CSVD is also on the rise. Clock genes have a significant impact on cognitive decline and dementia. Furthermore, the pattern of DNA methylation in clock genes is strongly associated with cognitive impairment. Thus, the aim of this study was to explore the connection between DNA promoter methylation of PER1 and CRY1 and cognitive dysfunction in patients with CSVD.

**Methods:**

We recruited patients with CSVD admitted to the Geriatrics Department of the Lianyungang Second People’s Hospital between March 2021 and June 2022. Based on their Mini-Mental State Examination score, patients were categorized into two groups: 65 cases with cognitive dysfunction and 36 cases with normal cognitive function. Clinical data, 24-h ambulatory blood pressure monitoring parameters, and CSVD total load scores were collected. Moreover, we employed methylation-specific PCR to analyze the peripheral blood promoter methylation levels of clock genes PER1 and CRY1 in all CSVD patients who were enrolled. Finally, we used binary logistic regression models to assess the association between the promoter methylation of clock genes (PER1 and CRY1) and cognitive dysfunction in patients with CSVD.

**Results:**

(1) A total of 101 individuals with CSVD were included in this study. There were no statistical differences between the two groups in baseline clinical data except MMSE and AD8 scores. (2) After B/H correction, the promoter methylation rate of PER1 was higher in the cognitive dysfunction group than that in the normal group, and the difference was statistically significant (adjusted *p* < 0.001). (3) There was no significant correlation between the promoter methylation rates of PER1 and CRY1 in peripheral blood and circadian rhythm of blood pressure (*p* > 0.05). (4) Binary logistic regression models showed that the influence of promoter methylation of PER1 and CRY1 on cognitive dysfunction were statistically significant in Model 1 (*p* < 0.001; *p* = 0.025), and it still existed after adjusting for confounding factors in Model 2. Patients with the promoter methylation of PER1 gene (*OR* = 16.565, 95%*CI*, 4.057–67.628; *p* < 0.001) and the promoter methylation of CRY1 gene (*OR* = 6.017, 95%*CI*, 1.290–28.069; *p* = 0.022) were at greater risk of cognitive dysfunction compared with those with unmethylated promoters of corresponding genes in Model 2.

**Conclusion:**

The promoter methylation rate of PER1 gene was higher in the cognitive dysfunction group among CSVD patients. And the hypermethylation of the promoters of clock genes PER1 and CRY1 may be involved in affecting cognitive dysfunction in patients with CSVD.

## Introduction

Cerebral small vessel disease (CSVD) is a heterogeneous disease caused by both genetic and vascular risk factors. It involves structural and functional abnormalities in small cerebral vessels and can cause variety of neuroimaging changes and neurological symptoms, including cognitive decline (Wardlaw et al., 2019; Zanon Zotin et al., 2021). Despite the lack of clear pathophysiological mechanisms, most patients with CSVD have similar brain parenchymal lesions, so the current clinical diagnosis of CSVD mainly relies on the indirect signs shown on the patients’ head MRI. Neuroimaging features of CSVD have been summarized as follows (Hu et al., 2021): white matter hyperintensity (WMH) of presumed vascular origin, lacunar infarction (LI), cerebral microbleed (CMB), perivascular space (PVS), recent small subcortical infarct (RSSI), and brain atrophy. Staals et al. (2014) constructed a CSVD total load model incorporating WMH, PVS, CMB, and LI to assess brain damage, which also improved the clinical diagnostic efficacy of CSVD. Given that CSVD is closely linked to aging, its prevalence is increasing in line with the global aging process (Li et al., 2020). CSVD has been identified as a key contributor to dementia by the National Institutes of Health (Gurol et al., 2020), implying that it is an important cause of cognitive dysfunction. Dementia has long posed a social and economic burden to countries around the world. China accounts for about 25% of the global dementia population (Jia et al., 2020). The status of the country with the largest number of dementia patients in the world brings great challenges to Chinese clinicians and family members. Therefore, there is an urgent need for preventing dementia and detecting cognitive dysfunction early.

24-h ambulatory blood pressure monitoring (ABPM) has been widely used in clinical practice and scientific research due to its ability to reflect blood pressure variation and circadian rhythm. As this application deepens, the relationship between blood pressure and cognitive function has gradually become a hot spot in the current research field. By monitoring ABPM in CSVD patients, Shim and Shin (2022) found that higher systolic blood pressure was linked to cognitive dysfunction and the severity of WMH in the elderly. In the same year, Tanaka and Hattori (2022) spotted that abnormal circadian rhythm of blood pressure could be related to cognitive impairment and poor outcomes in α-synucleinopathies. In addition, we also observed in our recent study that the disturbance of circadian rhythm of blood pressure might affect the cognitive function of CSVD patients, especially in non-dipper and reverse-dipper types (Xu et al., 2023).

Circadian rhythm of blood pressure, an essential aspect of circadian rhythms, is likewise regulated by the circadian clock. On a molecular level, this clock involves a complex set of autoregulatory transcription-translation feedback loops. And circadian rhythm mainly relies on the expression and function of clock genes and their encoded proteins involved in the feedback loops (Morris et al., 2016; Ramos-Lopez et al., 2018). The primary feedback loop consists of core clock components such as brain and muscle ARNT-like 1 (BMAL1, also known as MOP3, encoded by ARNTL), circadian locomotor output cycles kaput (CLOCK, encoded by CLOCK), period (PER, encoded by PER1, PER2, PER3), and cryptochrome (CRY, encoded by CRY1, CRY2; Kim and Lazar, 2020). The main circadian changes observed in aging include the decrease in amplitude and the advance of daily rhythm phase (de Souza Teixeira et al., 2020). Several aging-related degenerative diseases have been found to be related to circadian rhythms, and alterations in the expression and function of clock genes could also affect these diseases such as Alzheimer’s disease (AD), Parkinson’s disease (PD), and osteoarthritis (Zhu et al., 2022). Moreover, circadian rhythms have a profound impact on cognitive function. In fact, studies have found that circadian rhythm disturbances could lead to cognitive decline and even dementia (Kyriacou and Hastings, 2010; Chellappa et al., 2018, 2019; Maiese, 2021). Similarly, clock genes also play a crucial role in cognitive loss and dementia (Maiese, 2019). Several animal studies have demonstrated that clock gene pathways appear to be altered in dementia (Bellanti et al., 2017; Petrasek et al., 2018). For instance, Bacalini et al. (2022) observed that the rs3027178 polymorphism in the PER1 gene was significantly associated with AD, and the rs3027178 exhibited similar genotypic frequencies in AD patients and the elderly. Oyegbami et al. (2017) found that the expression of clock genes PER1, PER2, CRY1, and CRY2 in the medullary/pons of control mice increased at night compared to the day, while the influence of CRY1 and CRY2 expression were weakened in the AD-related transgenic mouse model (APPswe/PS1dE9). Furthermore, circadian rhythms of blood pressure and elevated blood pressure, as vital components of circadian rhythms, have also been found to be related to clock genes. Solocinski et al. (2017), in their knockout experiments on mice, found that blood pressure in wild-type mice was not affected by a high salt diet plus mineralocorticoid, whereas PER1 knockout mice exposed to this influence exhibited significantly increased mean arterial pressure and resulted in a non-dipper phenotype, suggesting that PER1 gene plays a critical role in regulating blood pressure. Kovanen et al. (2015) also supported the association of CRY1 with arterial hypertension and elevated blood pressure.

Researchers in the biomedical field have found that genetic analysis can help clarify the relationship between epigenetics, the circadian clock, and cognition. Methylation of the CpG island region of DNA promoter is one of the major regulatory mechanisms of gene transcription. Several studies have found that DNA methylation of clock genes is closely associated with cognitive impairment (Cronin et al., 2017; Kim et al., 2022). But until now, we have not found any studies focusing on the relationship between DNA methylation of clock genes and cognitive dysfunction in CSVD patients. In light of the above background, the aim of this study was to investigate the association of DNA promoter region methylation levels of PER1 and CRY1 with cognitive dysfunction in CSVD patients.

## Materials and methods

### Study population

**Figure 1:**
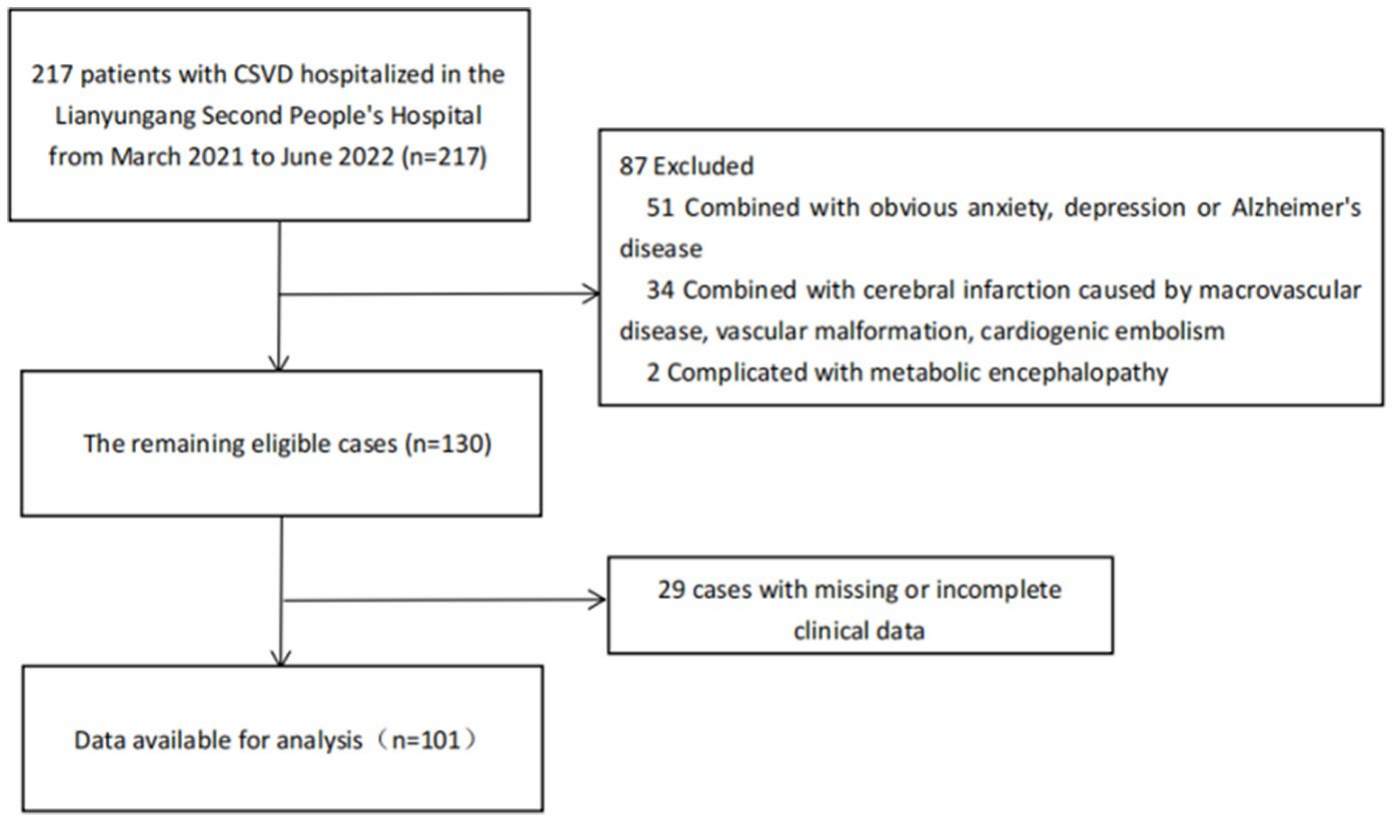
Flow diagram of included and excluded patients. CSVD, cerebral small vessel disease.

From March 2021 to June 2022, there were 217 individuals admitted to the Geriatrics Department of the Lianyungang Second People’s Hospital. Patients over 18 years old diagnosed with CSVD according to the Chinese consensus on diagnosis and therapy of cerebral small vessel disease 2021 (Hu et al., 2021) were included in this study. The subjects meeting the following conditions were excluded: (1) combined with obvious anxiety, depression or AD affecting cognitive function; (2) combined with cerebral infarction caused by macrovascular disease, vascular malformation, cardiogenic embolism, and other factors; (3) WMH caused by other diseases such as multiple sclerosis, metabolic, or toxic encephalopathy; (4) unable to cooperate with the head MRI examination; (5) unable to complete the Mini-Mental State Examination (MMSE) due to deafness, aphasia, agnosia, and other reasons; (6) suffering from severe insomnia; and (7) missing or incomplete clinical data. After exclusion, 101 patients with CSVD were enrolled in the study. Due to the epidemic prevention and control phase in China during the recruitment period, all patients were not infected with severe acute respiratory syndrome coronavirus 2 (SARS-CoV-2) when enrolled, and 37.6% of them had received at least one dose of vaccine. Among them, 65 patients with cognitive impairment were classified as the “cognitive dysfunction group” by MMSE score, and the remaining 36 patients were classified as the “normal group,” as shown in Figure 1. This study was approved by the Ethics Committee of Lianyungang Second People’s Hospital and registered in the Chinese Clinical Trial Registry (ChiCTR2000041152). Written informed consent was obtained from all participants.

### Cognitive function assessment

Mini-Mental State Examination was used to evaluate the cognitive function of enrolled patients, including five main components: orientation (0–10 points), memory (0–3 points), attention and numeracy (0–5 points), recall ability (0–3 points), and language ability (0–9 points). The total score was 30 points. The higher the score was, the better the cognitive function was. The normal threshold of MMSE score was defined as (Wang and Zhang, 1989): illiterate (uneducated) >17 points, primary school (years of education ≤6) >20 points, junior high school or above (years of education >6) >24 points. According to the MMSE score, the patients were divided into cognitive dysfunction group (< normal threshold) and normal group (> normal threshold).

### Extraction of DNA

A volume of 5 mL venous blood was collected from subjects at about 9 am after fasting for 12 h and placed into EDTA anticoagulation tubes, and then being mixed for 10–20 min and stored at −80°C. The Genomic DNA was extracted in strict accordance with the instructions of TIANamp Genomic DNA Kit (Servicebio, China). Then, about 1 μg of each genomic DNA sample was removed and modified according to the requirements of the modification kit Zymo DNA Methylation Kit (ZYMO, United States).

### The design of primers

Next, we analyzed the promoter sequences of clock genes PER1 and CRY1 at Methprimer online^1^ and designed methylation-specific PCR (MSP) primers for two clock genes. The designed primer sequences were shown in Table 1.

**Table 1:** The primers used for methylation analysis in the promoter of clock genes PER1 and CRY1.

Gene name	Primer	Sequence (5′–3′)	Amplicon/bp
PER1	Mf	GAGATTTTTAGTTAATCGGGGGC	228
	Mr	CAACGATCCGACTCAAAAACG	
	Uf	GAAGAGATTTTTAGTTAATTGGGGGT	236
	Ur	AATAACAACAATCCAACTCAAAAACA	
CRY1	Mf	ACGTGAGGTGTCGGTGGTTAC	220
	Mr	AAATAAACCCCTATCGACGACG	
	Uf	GAAATGTGAGGTGTTGGTGGTTAT	226
	Ur	ATCAACAACACTATCTCTCAACCCA	

### Methylation-specific PCR

In the 50 μL MSP reaction system, the disulfite modified genomic DNA template was 1.5 μL, the 2 × Taq PCR Master Mix was 25 μL, the upstream primer was 0.8 μL, the downstream primer was 0.8 μL, and ultra-pure water was added to make up the remaining volume. The mixture was then placed on a PCR instrument (Catalog: ETC811, EASTWIN, China) for amplification. The specific reaction conditions were as follows: pre-denaturation at 95°C for 5 min, denaturation at 95°C for 30 s, annealing at 55°C for 30 s, extension at 72°C for 30 s, amplification for 40 cycles, and then extension at 72°C for 5 min, and finally cooling at 16°C for 2 min.

**Figure 2:**
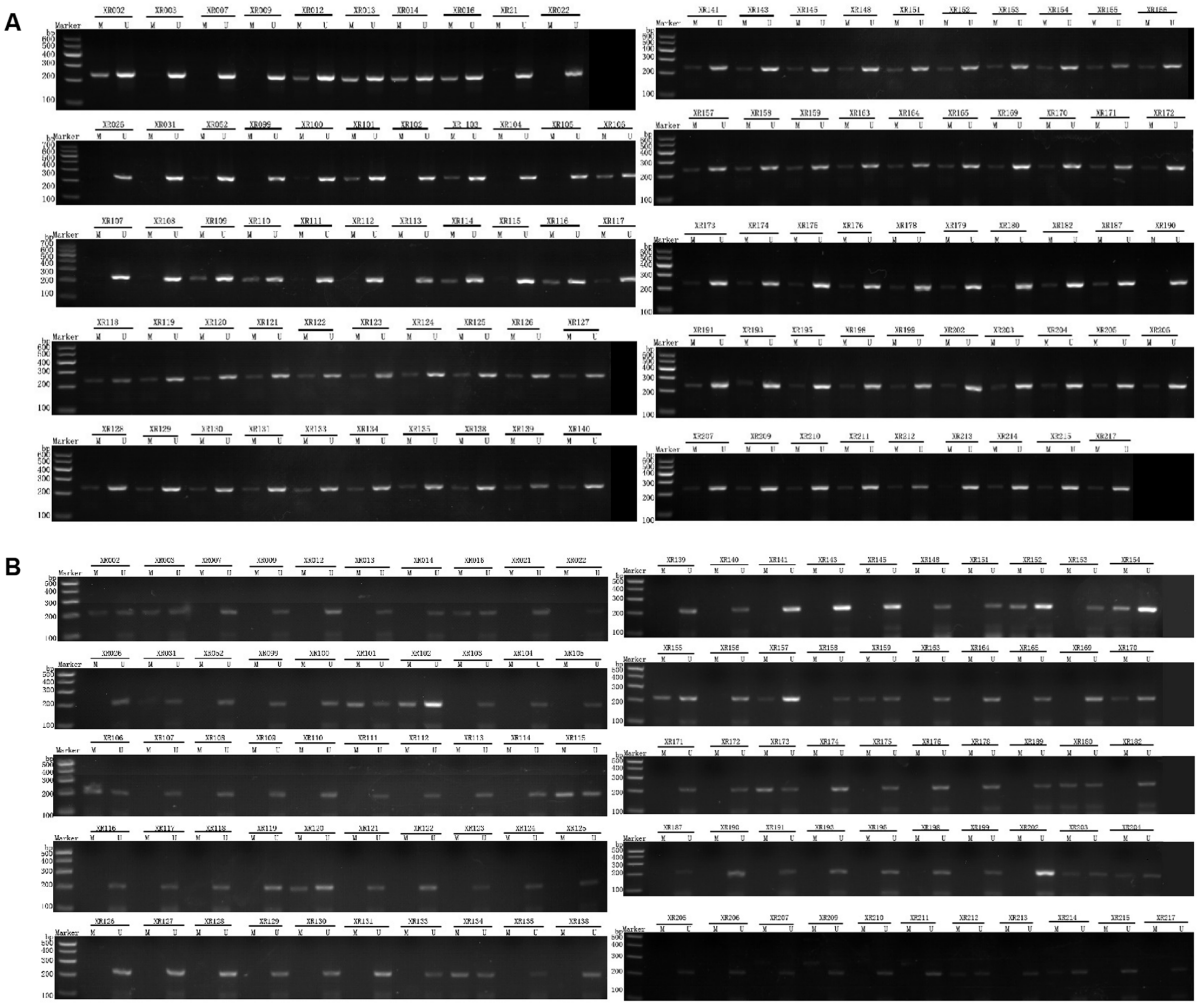
Electrophoretic diagram of test samples. **(A)** PER1; **(B)** CRY1. After PCR amplification, the amplified products were subjected to 2.0% agarose gel electrophoresis, and the images were recorded by the gel image analysis system. The presence of partial methylation in the DNA promoter region was represented by “M”, while the absence of methylation was represented by “U”.

Upon completion of the PCR amplification, the amplified products were subjected to 2.0% agarose gel electrophoresis, and the images were recorded by the gel image analysis system. The presence of partial methylation in the DNA promoter region was represented by “M’’ and the absence of methylation was represented by “U’’, as shown in Figure 2.

### Ambulatory blood pressure monitoring

All patients were required to have a successful 24-h ABPM (MedLifeKC-2820, China) within 48 h after diagnosed with CSVD, measured by a trained nurse. In the equipment used, blood pressure was measured every 30 min during the day (7:00–21:00) and every 60 min during the night (21:00–7:00). Each monitoring lasted for more than 25 h to ensure complete recording for 24 h, and eligibility was defined as valid data >80% for 24 h. Patients were asked to get enough rest or sleep at night and maintain normal activities during the day. Daily activities, sleep and wake times needed to be recorded in a diary. After wearing the device for at least 24 h, the device was removed and its data downloaded for analysis. Then relevant blood pressure parameters were collected, such as 24-h mean systolic blood pressure, 24-h mean diastolic blood pressure, 24-h mean standard deviation of systolic blood pressure, 24-h mean standard deviation of diastolic blood pressure, daytime mean systolic blood pressure (dmSBP), daytime mean diastolic blood pressure (dmDBP), nighttime mean systolic blood pressure (nmSBP), and nighttime mean diastolic blood pressure (nmDBP). And then we calculated the ratios of night systolic and diastolic blood pressure reduction separately: the ratio of night systolic blood pressure reduction = (dmSBP-nmSBP)÷dmSBP × 100%, the ratio of night diastolic blood pressure reduction = (dmDBP-nmDBP) ÷ dmDBP × 100%. We defined the ratio of night systolic blood pressure reduction as the ratio of night blood pressure reduction (ΔMBP) when the circadian rhythms of blood pressure shown by the ratio of night systolic and diastolic blood pressure reduction were inconsistent. Next, the circadian rhythms of blood pressure were classified according to ΔMBP: extreme-dippers (≥ 20%); dippers (10–20%); non-dippers (0–10%); and risers (< 0%; Kario et al., 2019). The classification of circadian rhythms of blood pressure was counted as an unordered categorical variable.

### CSVD total load score evaluation

The head MRI data of all subjects were acquired using a MAGNETOM Spectra 3.0 T magnetic resonance scanner with 16-channel coils. The sequences included: T1-weighted sequences (T1WI): TR 1,750 ms, TE 21.8 ms; T2-weighted image (T2WI): TR 3,598 ms, TE 107.3 ms; fluid-attenuated inversion recovery (FLAIR): TR 8,400 ms, TE 87 ms; diffusion weighted imaging (DWI): TR 6,000 ms, TE 73.5 ms; and susceptibility-weighted imaging (SWI): TR 37.5 ms, TE 22.9 ms, FOV 240 mm × 240 mm, and matrix 416 × 320. The CSVD total load score in this study included the four most classic imaging manifestations of WMH, LI, CMB, and PVS, with a total score of 0–4. It could reflect the severity of CSVD and was used as an ordered categorical variable in this study. A score of 0–1 was defined as mild, 2 as moderate, and 3–4 as severe (Xu et al., 2021). The evaluation was conducted independently by an experienced neurologist who had no other clinical information about the subjects.

### Statistical analysis

Statistical analyzes were performed using the SPSS software (IBM SPSS Statistics for Windows, version 26.0; IBM Corp., Armonk, NY, United States) and graphics were drawn by GraphPad Software (GraphPad Prism for Windows, version 9.0.0; San Diego, CA, United States). Kolmogorov–Smirnov test was used to evaluate the normality of numerical variables. Continuous variables of normal distribution were analyzed by independent sample T-test and expressed as mean ± SD. Continuous variables of skewed distribution were analyzed by Mann–Whitney U test and described by median and quartile range (IQR). And categorical variables were compared using the Chi-square test or Fisher’s exact test. We also used binary logistic regression models to assess the association between the promoter methylation of clock genes (PER1 and CRY1) and cognitive dysfunction in patients with CSVD. Due to the number of statistical analyzes we did, bilateral *p* values were adjusted according to the method of Benjamini-Hochberg (B/H) to control the false discovery rate (FDR). If the corresponding B/H-adjusted *p* value was lower than 0.05, the difference was considered to be statistically significant.

## Results

### Clinical characteristics of the participants

From May 2021 to June 2022, a total of 101 patients (55.4% male, average age 70 years) were included in the study. The clinical parameters of the subjects were shown in Table 2. It was observed that after B/H correction, there were no statistical differences between the two groups in baseline clinical data except MMSE and AD8 scores.

**Table 2:** Baseline characteristics of the participants.

	Overall (*N* = 101)	Cognitive dysfunction group (*N* = 65)	Normal group (*N* = 36)	Unadjusted *p* value	B/H-adjusted *p* value
Clinical parameters					
Age, Mean (SD)—year	70.01 ± 13.01	72.46 ± 12.75	65.58 ± 12.47	0.010	0.109
Gender—no. (%)				0.394	0.674
Male	56 (55.4%)	34 (52.3%)	22 (61.1%)		
Female	45 (44.6%)	31 (47.7%)	14 (38.9%)		
Educational level—no. (%)				0.029	0.220
Illiteracy	15 (14.9%)	12 (18.5%)	3 (8.3%)		
Primary school education	23 (22.8%)	14 (21.5%)	9 (25.0%)		
Junior high school education	33 (32.7%)	26 (40.0%)	7 (19.4%)		
High School education	22 (21.8%)	10 (15.4%)	12 (33.3%)		
Undergraduate college	8 (7.9%)	3 (4.6%)	5 (13.9%)		
AD8 score, Median (IQR)	2.00 (1.00, 4.00)	4.00 (2.00, 5.00)	1.00 (0.00, 1.75)	<0.001	<0.001
MMSE score, Median (IQR)	20.00 (11.00, 26.5.00)	12.00 (7.50, 19.00)	28.00 (26.00, 28.75)	<0.001	<0.001
BMI, Mean (SD)—kg/m^2^	25.63 ± 3.53	25.71 ± 3.54	25.50 ± 3.56	0.782	0.922
SBP, Mean (SD)—mmHg	144.95 ± 20.96	146.11 ± 21.96	142.86 ± 19.15	0.459	0.737
DBP, Mean (SD)—mmHg	85.14 ± 16.22	85.97 ± 16.91	83.64 ± 15.00	0.492	0.745
Glucose, Mean (SD)—mmol/L	8.25 ± 3.51	7.99 ± 3.65	8.15 ± 3.26	0.837	0.944
Medical history					
Hypertension—no. (%)	72 (71.3%)	51 (78.5%)	21 (58.3%)	0.032	0.212
Diabetes mellitus—no. (%)	37 (36.6%)	28 (43.1%)	9 (25.0%)	0.071	0.251
TIA or stroke—no. (%)	41 (40.6%)	30 (46.2%)	11 (30.6%)	0.126	0.334
Cardiac disease—no. (%)	37 (36.6%)	23 (35.4%)	14 (38.9%)	0.726	0.916
Smoking—no. (%)	15 (14.9%)	9 (13.8%)	6 (16.7%)	0.703	0.909
Drinking—no. (%)	18 (17.8%)	11 (16.9%)	7 (19.4%)	0.751	0.905
Antiplatelet drugs—no. (%)	50 (49.5%)	33 (50.8%)	17 (47.2%)	0.733	0.903
Antihypertensive drugs—no. (%)	58 (57.4%)	39 (60.0%)	19 (52.8%)	0.482	0.751
Hypoglycemic drugs—no. (%)	26 (25.7%)	14 (21.5%)	12 (33.3%)	0.194	0.467
Antihyperlipidemics—no. (%)	54 (53.5%)	35 (53.8%)	19 (52.8%)	0.918	0.973
Laboratory indicators					
WBC, Mean (SD)—10^9/L	6.60 ± 1.99	6.77 ± 2.08	6.29 ± 1.78	0.258	0.526
RBC, Mean (SD)—10^12/L	4.44 ± 0.52	4.41 ± 0.55	4.49 ± 0.48	0.512	0.754
HGB, Mean (SD)—g/L	136.57 ± 17.20	134.58 ± 17.67	140.35 ± 15.82	0.113	0.334
PLT, Mean (SD)—10^9/L	216.60 ± 77.47	226.45 ± 87.47	197.76 ± 49.28	0.080	0.266
NEUT, Median (IQR)—10^9/L	3.97 (3.01, 5.17)	4.00 (3.02, 5.06)	3.34 (2.75, 4.25)	0.082	0.256
LY, Median (IQR)—10^9/L	1.75 (1.25, 2.18)	1.76 (1.23, 2.31)	1.72 (1.13, 2.22)	0.883	0.955
ALT, Median (IQR)—U/L	24.00 (19.00, 35.00)	20.00 (14.00, 34.00)	27.00 (23.50, 38.50)	0.026	0.230
AST, Median (IQR)—U/L	26.00 (22.00, 31.00)	25.00 (22.00, 31.00)	26.00 (22.00, 30.00)	0.950	0.968
BUN, Median (IQR)—mmol/L	6.40 (5.20, 8.70)	6.00 (4.75, 8.70)	6.20 (5.40, 7.15)	0.211	0.486
Cr, Median (IQR)—μmol/L	67.00 (55.00, 87.00)	63.00 (53.50, 92.00)	67.00 (54.50, 83.00)	0.241	0.532
UA, Median (IQR)—μmol/L	312 (258.00, 388.00)	296.00 (248.50, 367.00)	312.00 (261.50, 404.50)	0.979	0.979
ALB, Median (IQR)—g/L	43.00 (38.50, 43.00)	41.00 (37.90, 44.45)	43.30 (39.70, 44.65)	0.044	0.212
TC, Mean (SD)—mmol/L	4.59 ± 1.31	4.69 ± 1.38	4.40 ± 1.19	0.291	0.551
LDL-C, Mean (SD)—mmol/L	2.87 ± 0.97	2.92 ± 1.00	2.79 ± 0.92	0.538	0.771
HDL-C, Mean (SD)—mmol/L	1.18 ± 0.29	1.19 ± 0.31	1.17 ± 0.25	0.683	0.906
TG, Median (IQR)—mmol/L	1.70 (1.19, 2.57)	1.52 (1.06, 2.29)	1.79 (1.30, 2.51)	0.298	0.545
Lp(a), Median (IQR)—mg/L	131.00 (63.50, 242.75)	185.00 (72.50, 324.50)	125.00 (50.00, 159.50)	0.118	0.328
FT4, Mean (SD)—pmol/L	12.24 ± 2.55	12.40 ± 2.56	11.93 ± 2.55	0.440	0.729
FT3, Mean (SD)—pmol/L	5.12 ± 0.87	5.09 ± 0.96	5.20 ± 0.66	0.590	0.802
TSH, Median (IQR)—uIU/mL	1.86 (1.28, 2.55)	1.61 (1.28, 2.21)	1.93 (1.05, 2.60)	0.939	0.976
D2-dimer, Median (IQR)—ng/ml	120.00 (70.50, 212.00)	131.50 (81.75, 262.75)	88.00 (59.00, 180.50)	0.047	0.193
INR, Mean (SD)	0.99 ± 0.10	0.98 ± 0.09	1.02 ± 0.11	0.042	0.224
PT, Mean (SD)—s	11.76 ± 1.22	11.59 ± 1.11	12.08 ± 1.37	0.059	0.222
FIB, Mean (SD)—g/L	4.10 ± 0.56	4.15 ± 0.59	4.02 ± 0.50	0.276	
CRP, Median (IQR)—mg/L	1.07 (0.40, 2.51)	1.70 (0.40, 5.75)	0.83 (0.41, 1.61)	0.187	0.472
Carotid artery ultrasonography					
CIMT, Mean (SD)—mm	0.83 ± 0.17	0.86 ± 0.18	0.78 ± 0.15	0.559	0.779
Carotid plaques				0.008	0.106
No—no. (%)	37 (36.6%)	24 (36.9%)	13 (36.1%)		
Stable plaque—no. (%)	44 (43.6%)	23 (35.4%)	21 (58.3%)		
Unstable plaque—no. (%)	20 (19.8%)	18 (27.7%)	2 (5.6%)		

SD, standard deviation; IQR, interquartile range; AD8, the Ascertain Dementia 8-item Questionnaire; MMSE, the Mini-Mental State Examination; BMI, body mass index; SBP, systolic blood pressure; DBP, diastolic blood pressure; WBC, the white blood cell count; RBC, red blood cell count; HGB, hemoglobin; PLT, platelet count; NEUT, neutrophil count; LY, lymphocyte count; ALT, alanine aminotransferase; AST, aspartate transaminase; BUN, urea nitrogen; Cr, creatinine; UA, uric acid; ALB, albumin; TC, total cholesterol; LDL-C, low-density lipoprotein cholesterol; HDL-C, high-density lipoprotein cholesterol; TG, total glyceride; Lp(a), Lipoprotein a; FT4, free thyroxine; FT3, free tri-iodothyronine; TSH, thyroid stimulation hormone; INR, international normalized ratio; PT, prothrombin time; FIB, fibrinogen; CRP, C-reactive protein; CIMT, carotid intima-media thickness; and B/H, Benjamini-Hochberg.

### Comparison of the promoter methylation of clock genes

**Figure 3:**
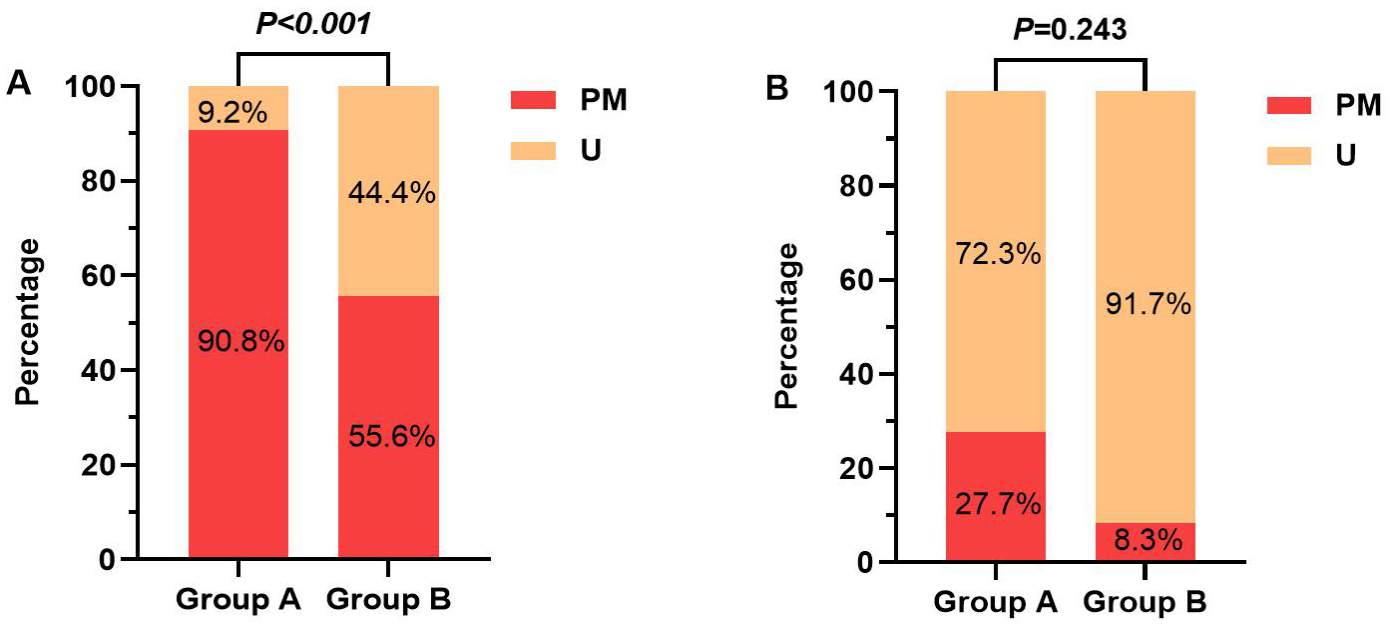
**(A)** The promoter methylation rates of PER1 gene between the two groups; **(B)** The promoter methylation rates of CRY1 gene between the two groups. Group A, Cognitive dysfunction group; Group B, Normal group; U, unmethylated; and PM, partially-methylated.

After B/H correction, the promoter methylation rate of the PER1 gene was higher in the cognitive dysfunction group than that in the normal group, and the difference was statistically significant (adjusted *p* < 0.001). However, there was no difference in the promoter methylation rate of the CRY1 gene between the two groups (adjusted *p* = 0.243), as shown in Table 3 and Figure 3. In terms of the amount of methylation, 59 cases (90.8%) were partially methylated in PER1 and 18 cases (27.7%) were partially methylated in CRY1 in 65 cases of cognitive dysfunction. While in the normal group, 20 cases (55.6%) had partial methylation of PER1 and three cases (8.3%) had partial methylation of CRY1.

**Table 3:** The amount and frequency of promoter methylation of clock genes in the two groups.

Gene name	U/PM	Overall (*N* = 101)	Cognitive dysfunction group (*N* = 65)	Normal group (*N* = 36)	*X* ^2^	Unadjusted *p* value	B/H-adjusted *p* value
PER1	PM	79 (78.2%)	59 (90.8%)	20 (55.6%)	16.862	<0.001	<0.001
	U	22 (21.8%)	6 (9.2%)	16 (44.4%)			
CRY1	PM	21 (20.8%)	18 (27.7%)	3 (8.3%)	4.162	0.041	0.243
	U	80 (79.2%)	47 (72.3%)	33 (91.7%)			

U, unmethylated; PM, partially-methylated; and B/H, Benjamini-Hochberg.

### Comparison of ABPM parameters

There were no statistically significant differences in ABPM parameters such as 24-h mean systolic blood pressure, 24-h mean diastolic blood pressure, 24-h mean standard deviation of systolic blood pressure, 24-h mean standard deviation of diastolic blood pressure, dmSBP, dmDBP, nmSBP, and nmDBP, and the classification of circadian rhythms of blood pressure between the two groups (*p* > 0.05), as shown in Table 4. In addition, no significant correlation was found between the promoter methylation rates of PER1 and CRY1 and the classification of circadian rhythms of blood pressure, as shown in Table 5.

**Table 4:** Comparison of ambulatory blood pressure parameters between the two groups.

Parameters	Overall (*n* = 71)	Cognitive dysfunction group (*n* = 46)	Normal group (*n* = 25)	*t*/*X*^2^	*p* value
24hmSBP, Mean (SD)—mmHg	140.44 ± 17.74	140.57 ± 19.09	140.19 ± 15.24	−0.104	0.917
24hmDBP, Mean (SD)—mmHg	78.88 ± 10.32	77.80 ± 10.76	80.82 ± 9.29	1.418	0.159
24hSBP-SD, Mean (SD)—mmHg	16.79 ± 4.05	16.94 ± 4.43	16.54 ± 3.30	−0.473	0.637
24hDBP-SD, Mean (SD)—mmHg	12.33 ± 2.82	12.06 ± 2.96	12.81 ± 2.50	1.281	0.203
dmSBP, Mean (SD)—mmHg	140.82 ± 18.47	140.83 ± 20.11	140.81 ± 15.32	−0.006	0.995
dmDBP, Mean (SD)—mmHg	79.55 ± 10.78	78.22 ± 11.23	81.95 ± 9.59	1.679	0.096
nmSBP, Mean (SD)—mmHg	138.36 ± 18.85	138.69 ± 19.52	137.78 ± 17.82	−0.231	0.818
nmDBP, Mean (SD)—mmHg	77.39 ± 10.40	76.76 ± 10.33	78.54 ± 10.58	0.822	0.413
Circadian rhythm of blood pressure—no. (%)				2.204	0.564
Dippers	13 (18.3%)	7 (15.2%)	6 (24.0%)	0.835	0.361
Extreme-dippers	1 (1.4%)	1 (2.2%)	0 (0.0%)	0.876	0.999
Non-dippers	28 (39.4%)	17 (37.0%)	11 (44.0%)	0.336	0.562
Reverse-dippers	29 (40.8%)	21 (45.7%)	8 (32.0%)	1.249	0.264

SD, standard deviation; 24hmSBP, 24-h mean systolic blood pressure; 24hmDBP, 24-h mean diastolic blood pressure; 24hSBP-SD, 24-h mean standard deviation of systolic blood pressure; 24hDBP-SD, 24-h mean standard deviation of diastolic blood pressure; dmSBP, daytime mean systolic blood pressure; dmDBP, daytime mean diastolic blood pressure; nmSBP, nighttime mean systolic blood pressure; and nmDBP, nighttime mean diastolic blood pressure.

**Table 5:** Relationship between the promoter methylation of clock genes and circadian rhythm of blood pressure.

Circadian rhythm of blood pressure—no. (%)	Overall (*N* = 71)	Methylation of PER1		*X* ^2^	*p* value	Methylation of CRY1		*X* ^2^	*p* value
		PM (*N* = 58)	U (*N* = 13)			PM (*N* = 14)	U (*N* = 57)		
Dippers	13 (18.3%)	10 (17.2%)	3 (23.1%)	0.898	0.877	2 (14.3%)	11 (19.3%)	0.709	0.999
Extreme-dippers	1 (1.4%)	1 (1.7%)	0 (0.0%)			0 (0.0%)	1 (1.8%)		
Non-dippers	28 (39.4%)	23 (39.7%)	5 (38.5%)			6 (42.9%)	22 (38.6%)		
Reverse-dippers	29 (40.8%)	24 (41.4%)	5 (38.5%)			6 (42.9%)	23 (40.4%)		

U, unmethylated; PM, partially-methylated.

### Comparison of CSVD total load scores

On the basis of the MRI data, we observed 40.6% cases with moderate–severe WMHs, 12.9% with enlarged perivascular spaces 2–4 Level, 13.9% with CMB, and 96.0% with LI. In these four separate imaging findings, there was no statistically significant difference between the two groups. Although it could be seen from Table 6 that, after combining these four imaging findings, more people in the cognitive dysfunction group were classified as severe compared with the normal group (*p* = 0.046). Unfortunately, after B/H correction, there was no statistically significant difference between the two groups (adjusted *p* = 0.203).

**Table 6:** Comparison of CSVD total load scores between the two groups.

Imaging findings on MRI—no. (%)	Overall (*N* = 101)	Cognitive dysfunction group (*N* = 65)	Normal group (*N* = 36)	*X* ^2^	Unadjusted *p* value	B/H-adjusted *p* value
Moderate–severe WMH	41 (40.6%)	26 (40.0%)	15 (41.7%)	0.027	0.870	0.961
EPVS 2–4 Level	13 (12.9%)	8 (2.3%)	5 (13.9%)	0.052	0.820	0.945
CMB	14 (13.9%)	11 (16.9%)	3 (8.3%)	0.803	0.370	0.654
LI	97 (96.0%)	64 (98.5%)	33 (91.7%)	1.310	0.252	0.534
Total CSVD burden				6.150	0.046	0.203
Mild	53 (52.5%)	35 (53.8%)	18 (50.0%)			
Moderate	33 (32.7%)	17 (26.2%)	16 (44.4%)			
Severe	15 (14.9%)	13 (20.0%)	2 (5.6%)			

WMH, white matter hyperintensities; EPVS, enlarged perivascular spaces; CMB, cerebral microbleeds; LI, lacunar infarcts; and B/H, Benjamini-Hochberg.

### Binary logistic regression analysis

We then used binary logistic regression models to investigate the relationship between the promoter methylation rates of clock genes and cognitive dysfunction in CSVD patients. The univariate analysis showed that the influence of promoter methylation of PER1 and CRY1 on cognitive dysfunction were all statistically significant in Model 1 (*p* < 0.001; *p* = 0.025), which still existed after we adjusted for confounding factors in Model 2. It was observed from Model 2 that patients with the promoter methylation of PER1 gene (*OR* = 16.565, 95%*CI*, 4.057–67.628; *p* < 0.001) and the promoter methylation of CRY1 gene (*OR* = 6.017, 95%*CI*, 1.290–28.069; *p* = 0.022) were at greater risk of cognitive dysfunction compared with those with unmethylated promoters of corresponding genes, as shown in Table 7.

**Table 7:** Association between the promoter methylation of clock genes and cognitive dysfunction.

		*B*	SE	WaldX^2^	*p* value	*OR*	95% *CI*
Model 1	Constant	−1.352	0.583	6.316	0.012		
	PER1 PM	2.191	0.583	14.107	<0.001	8.945	2.851–28.063
	CRY1 PM	1.653	0.739	5.005	0.025	5.221	1.227–22.211
Model 2	Constant	−6.021	1.818	10.972	0.001		
	PER1 PM	2.807	0.718	15.298	<0.001	16.565	4.057–67.628
	CRY1 PM	1.795	0.786	5.216	0.022	6.017	1.290–28.069

B, regression coefficient; SE, standard error; WaldX2, chi-square value; OR, odds ratio; CI, confidence interval; and PM, partially-methylated.

Model 1: unadjusted.

Model 2: adjusted for age, history of hypertension, history of diabetes mellitus, and the CSVD total load score.

## Discussion

In the present study, we demonstrated that, in patients with CSVD, the methylation level in the promoter regions of the PER1 gene was higher than that in the normal group (*p* < 0.001). To the best of our knowledge, this is the first study to investigate the relationship between the promoter methylation of peripheral blood clock genes PER1 and CRY1 and cognitive dysfunction in patients with CSVD patients.

Circadian rhythm disturbances, such as sleep disorders, are very common in aging and are present in many neurodegenerative diseases (Kondratova and Kondratov, 2012). As mentioned in the introduction, the circadian clock regulates circadian rhythms of the organism. Therefore, at the level of molecular structure of the circadian clock, the mechanism of circadian rhythm disturbances might be as follows (Wu and Swaab, 2007): (1) A decreased input to the suprachiasmatic nucleus (SCN); (2) Alterations in the SCN; (3) Changes in the pineal gland, melatonin, and its receptors. Among them, clock genes PER and CRY aroused our interest as the important part of the core clock components with their crucial roles in both the SCN and peripheral clock.

Previous studies believed that the influence of circadian rhythm on long-term memory originated from the disorder within the SCN, which then drove alterations in peripheral structures involved in memory formation. However, Kwapis et al. (2018) found that reducing PER1 expression directly in the dorsal hippocampus could impair long-term memory in young mice whereas local overexpression of PER1 in the dorsal hippocampus could improve memory in aging mice. Their conclusions challenged conventional assumptions and demonstrated that PER1 plays a key role within local memory structures that alters memory formation, independent of its function in the SCN. In the same year, Brzezinski et al. (2018) found that CRY1, CRY2, PER1, and PER2 and other clock genes were all expressed in cultured human luteinized granulosa cells, and the expression in aged female cells generally showed a downward trend, including PER1. In addition, since the potential link between circadian rhythm disturbances and the development of AD has not been clearly established, Niu et al. (2022) attempted to establish the link through chronic sleep deprivation (CSD) in a recent study. Their results showed that CSD impaired learning and memory in AD mice and further accelerated AD progression. Also, CSD induced abnormal expressions of CRY1, CLOCK, and BMAL1 in the circadian rhythm-related nucleus of experimental mice, which were more significant in AD mice. In conclusion, these previous studies were sufficient to convince us that clock genes PER1 and CRY1 were associated with aging and cognitive dysfunction, which is also the reason why we chose these two genes. Our findings suggested that the methylation of these two genes in the promoter region of peripheral blood might be useful markers for cognitive dysfunction in CSVD patients.

In recent years, there has been increasing interest in the relationship between epigenetics and cognitive function. DNA methylation is one of the most characteristic epigenetic modifications, which can affect the activity of a DNA segment without changing the sequence (Prado et al., 2021), so it has long been favored by researchers. Previous studies have shown that epigenetics, especially DNA methylation, plays a very important role in aging (Unnikrishnan et al., 2019). A study that investigated methylation changes in 217 non-pathologic human tissue samples showed that methylation changes were significantly correlated with aging and various environmental exposures such as smoking (Christensen et al., 2009). One of the primary end-points related to aging is the loss of neuronal function, which further leads to impaired memory and cognitive function (Yang et al., 2019). In fact, altered DNA methylation has been observed to be associated with age-related memory loss in animal studies (Ianov et al., 2017). As the most famous age-related diseases, previous studies have also attempted to investigate the underlying mechanisms of abnormal expression of clock genes in diseases such as PD and AD from the perspective of DNA methylation. Lin et al. (2012) detected the methylation levels of the promoters of seven major human clock genes in order to investigate the underlying mechanisms of the altered expression of clock genes in leukocytes from PD patients, and then found that methylation could only be detected in the CRY1 and NPAS2 promoters and the methylation frequency of NPAS2 promoter was significantly reduced in PD patients. Di Francesco et al. (2015) found an increase in global DNA methylation in late-onset AD peripheral blood mononuclear cells compared to healthy controls, and associated with worse cognitive performances. Besides, many studies have found that the promoter methylation of clock genes could also be detected in dementia. Liu et al. (2008) included 80 dementia patients and 80 age- and gender-matched controls to assess the promoter methylation status of nine clock genes in dementia, and observed that only the PER1 and CRY1 promoter CpG islands were methylated in dementia patients (7/80), while none of the other clock genes involved were methylated. Based on this, we initially proposed the hypothesis that the cognitive dysfunction group of CSVD patients would have higher promoter methylation rates of PER1 and CRY1, further suggesting lower gene expression. In fact, our results appeared to be consistent with Liu et al. (2008), but the promoter methylation rates of the two genes in our results were much higher. Given that DNA methylation has been reported to change with aging in previous studies, we suspected that this might be related to the older age of the patients we enrolled (with the average age of 70). At the same time, this study was conducted on patients with CSVD, which is known as one of age-related diseases, so we guessed that our results were relatively reasonable. Our results showed that there was no significant difference in the promoter methylation rate of CRY1 between the two groups after B/H correction. However, referring to the positive results of previous studies, we still included them in the subsequent binary logistic regression analysis. Through analysis, we also found that hypermethylation of the promoters of clock genes PER1 and CRY1 may be involved in affecting cognitive dysfunction in patients with CSVD.

It is worth noting that, to obtain test samples easily, we measured DNA methylation in peripheral blood cells rather than in other metabolically active tissues such as muscle, liver, or adipocytes. Many previous studies have been conducted on peripheral blood cells related to clock genes, and the methylation characteristics in blood cells have been consistently reflected in other tissues (Crujeiras et al., 2017; Ramos-Lopez et al., 2018).

This study had some limitations. First, the overnight polysomnography and the Pittsburgh Sleep Quality Index (PSQI) were not administered to assess patients’ sleep quality and duration of the night in detail, which may affect DNA methylation. Second, we only measured the methylation level of clock genes at one point in time. Third, as a single-center study with a small sample size, there was a certain selection bias. In addition, as it was a cross-sectional study, we could not explain the causal relationship and specific mechanism between the promoter methylation of PER1, CRY1 and cognitive impairment in CSVD patients.

## Conclusion

The promoter methylation rate of PER1 gene was higher in the cognitive dysfunction group among CSVD patients. And the hypermethylation of the promoters of clock genes PER1 and CRY1 may be involved in affecting cognitive dysfunction in patients with CSVD.

## Data availability statement

The raw data supporting the conclusions of this article will be made available by the authors, without undue reservation.

## Ethics statement

The studies involving human participants were reviewed and approved by the Ethics Committee of Lianyungang Second People’s Hospital. The patients/participants provided their written informed consent to participate in this study.

## Author contributions

YX and XS conceived and designed the research. YX and YW analyzed the data and drafted the manuscript. YX, YJ, ML, WZ, ZG, and ZS collected the data and performed the research. ZS and XS reviewed and edited the manuscript. All authors contributed to the article and approved the submitted version.

## Funding

This study was supported by Open-end Funds of Jiangsu Key Laboratory of Marine Pharmaceutical Compound Screening (HY202203), Science and Technology Project of Lianyungang Health Commission (202024), Scientific Research Project of Bengbu Medical College (2020byzd341), Jiangsu Provincial Geriatric Health Research Grant Project (LD2021034 and LR2021049), Jiangsu Province Postgraduate Practice Innovation Program (SJCX21_1726), and Jiangsu Province “Six One Project” Top Talent to be funded project (LGY2019062).

## Conflict of interest

The authors declare that the research was conducted in the absence of any commercial or financial relationships that could be construed as a potential conflict of interest.

## Publisher’s note

All claims expressed in this article are solely those of the authors and do not necessarily represent those of their affiliated organizations, or those of the publisher, the editors and the reviewers. Any product that may be evaluated in this article, or claim that may be made by its manufacturer, is not guaranteed or endorsed by the publisher.

## Footnotes

^1^ http://www.urogene.org/cgi-bin/methprimer/methprimer.cgi
